# Combined Ravitch and Nuss procedure for patients with severe pectus excavatum: technique and initial results

**DOI:** 10.1093/jscr/rjad576

**Published:** 2023-10-31

**Authors:** Kyohei Masai, Yu Okubo, Kaoru Kaseda, Tomoyuki Hishida, Keisuke Asakura

**Affiliations:** Division of Thoracic Surgery, Department of Surgery, Keio University School of Medicine, 35 Shinanomachi, Shinjuku-ku Tokyo 160-8582, Japan; Division of Thoracic Surgery, Department of Surgery, Keio University School of Medicine, 35 Shinanomachi, Shinjuku-ku Tokyo 160-8582, Japan; Division of Thoracic Surgery, Department of Surgery, Keio University School of Medicine, 35 Shinanomachi, Shinjuku-ku Tokyo 160-8582, Japan; Division of Thoracic Surgery, Department of Surgery, Keio University School of Medicine, 35 Shinanomachi, Shinjuku-ku Tokyo 160-8582, Japan; Division of Thoracic Surgery, Department of Surgery, Keio University School of Medicine, 35 Shinanomachi, Shinjuku-ku Tokyo 160-8582, Japan

**Keywords:** pectus excavatum, Nuss procedure, Ravitch procedure, CRN procedure

## Abstract

The Nuss procedure for pectus excavatum (PE) is both less invasive and very simple compared to the Ravitch procedure. However, it may be difficult to perform the Nuss procedure in cases of severe PE. Therefore, we developed a Combined Ravitch and Nuss (CRN) procedure and examined its effectiveness in patients with severe PE. Nine patients with severe PE underwent the CRN procedure. Data on patient characteristics and perioperative results were collected retrospectively. The median Haller index (HI) was 15.4 (range, 6.3–29.3). No significant intraoperative adverse events were noted. Postoperatively, marked improvements in HI were seen in all cases (3.29, range, 2.72–4.96). Two surgical site infections on the shallow layer and one wound seroma occurred. No recurrences were observed during the observation period. Our novel CRN procedure is useful for achieving adequate and sustainable sternal elevation with less invasiveness for patients with severe PE.

## Introduction

Pectus excavatum (PE) is the most common congenital chest wall deformity. In 1949, the Ravitch procedure for treating PE was developed [1]. This procedure was useful for elevating the chest wall, it was also highly invasive. In contrast, Nuss *et al.* [2] reported a completely different, minimally invasive technique for PE correction in 1998. Subsequently, perioperative management for PE improved dramatically. The Nuss procedure has since become the standard procedure for treating PE.

The Nuss procedure involves the insertion of curved metal bars behind the sternum to push it up to a normal position. Although it is minimally invasive, this procedure has some technical issues. The most important issue is the challenge of manipulating the mediastinum in a severely recessed sternum. In addition, excessive precordial elevation may cause adverse intraoperative events such as rib fracture. Thus, in 2017, we developed a combined Ravitch and Nuss (CRN) procedure as a new technique for treating patients with severe PE. In this study, we examined the effectiveness of the CRN procedure in nine cases of severe PE.

## Case series

### Patients and methods

In a 2-year period (2017–2019), we treated nine patients with severe PE using the CRN procedure. The indications for CRN approach were (a) severe PE with a Haller index on computed tomography scans (HI–CT) of >10 sinking [3], (b) stenosis of the central pulmonary blood vessel and bronchus causing cardiopulmonary dysfunction, and (c) recurrent cases with severe deformity after surgery for PE. The clinical data of the patients were retrospectively reviewed.

### Operative technique

**Figure 1:**
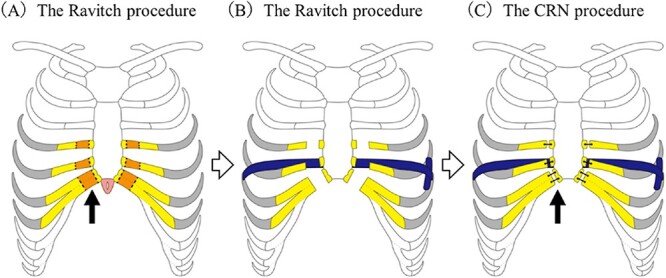
Operative technique for the combined Ravitch and Nuss procedure. (A) The Ravitch procedure. The deformed bilateral cartilages were transected from the third or fourth to the seventh costal cartilage junctions. (arrow) After these procedures, the recessed sternum could be easily raised. (B) The Nuss procedure. A titanium pectus bar was passed across the mediastinum under the transected sternum and rotated 180° to stabilize the anterior chest wall. (C) Final step. After the edge of the costal cartilage was trimmed, suturing to the edge of the costal cartilage and sternum was done using surgical wire or thread sutures (arrow).

All nine patients underwent the CRN procedure (Fig. 1). The CRN procedure is composed of three steps. First, the severely depressed portion of the costal cartilage is transected. Second, the sternum is stabilized using pectus bars and last step, the anterior chest wall is then reconstructed.

The procedure is as follows: Mercedes incisions were made in the most recessed portion of the chest. The pectoralis and rectus muscles were mobilized to expose the deformed costal cartilages. Next, bilateral ribs from the third or fourth to the seventh costal cartilage junctions were disconnected at the adjacent sternum. At that time, the bilateral internal mammary arteries were carefully preserved. According to the original Ravitch procedure, in one case, transverse osteotomy of the sternum was performed at the level of the third rib. Although two cases did not undergo transverse osteotomy, a transverse slit was made in the upper part of the sternum for correction. In the remaining cases, neither a transverse osteotomy nor a transverse slit was made. With dissection of the bilateral ribs costal cartilage, the recessed sternum developed increased mobility (part of the Ravitch procedure).

After the sternum was mobilized, the Nuss procedure was performed. A thoracoscope was used to view the safe placement of the introducer below the sternum. A titanium pectus bar was passed across the mediastinum under the reconstructed sternum and then rotated 180 degrees to stabilize the anterior chest wall. Since the cut end of the costal cartilages protruded about 1–2 cm due to elevation of the anterior chest wall, it was necessary to trim the edge of costal cartilages to flatten the anterior chest cartilage wall. After this trimming, we performed re-suturing of the costal cartilage edge and sternum using surgical wire or thread sutures.

## Results

**Figure 2:**
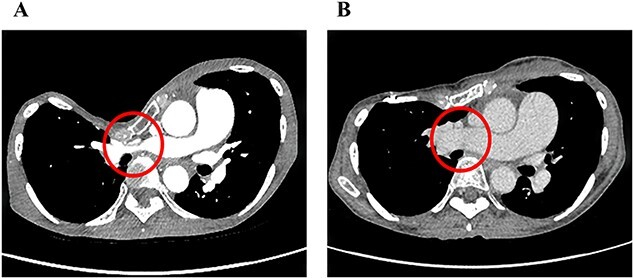
Pre- (A) and postoperative (B) chest CT findings of severe PE with stenosis of the right main pulmonary artery. The diameter of the right main pulmonary artery increased from 5 to 20 mm after the CRN procedure.

The clinical characteristics of the nine cases are summarized in Table 1. Median follow-up since our surgery is 53 months. The median age was 28 years (range, 16–74). These patients were categorized into two groups: eight patients with primary PE and one with PE recurrence after the Nuss procedure. Six had subjective symptoms such as palpitations and respiratory discomfort. Only one case had significant findings on preoperative echocardiography suggestive of pulmonary hypertension. In radiological findings, the HI–CT for PE was 15.4 (range, 6.3–29.3). One case had severe stenosis of the right main pulmonary artery due to sternal depression (Fig. 2A). The perioperative results of the CRN procedure are summarized in Table 2. Intraoperatively, no significant adverse events were noted. The median operation time was 223 min (range, 154–307 min) and the median volume of blood loss was 165 g (range, 10–904 g). The median length of hospital stay was 8 days (range, 6–37 days). Marked improvements in the HI–CT were seen in all cases (3.29, range, 2.72–4.96). In one case with stenosis of the right main pulmonary artery, not only did HI–CT improve, but also the diameter of the right main pulmonary artery increased from 5 to 20 mm after PE correction (Fig. 2B). Regarding postoperative complications, surgical site infections of the shallow layers were observed in two cases. No patient required re-operation due to adverse events.

**Table 1:** Clinical characteristics of nine cases with severe PE.

Case	Age (years)	Symptom	Medical history	Family history	Smoking history	UCG	ECG	%VC	FEV1.0%	HI-CT	Sternal torsion	Scoliosis
1	65	Chronic respiratory failure	DM	−	−	PH	RVH	41.9	72.7	29.3	54.3	−
2	28	Dyspnea	None	+	−	None	None	75.9	91.6	19.1	12.3	−
3	26	Dyspnea	None	−	−	None	LAL	65.3	67	11.6	2.2	+
4	74	Chronic respiratory failure	Af	+	+	None	Af	65.3	71.3	12.5	36.3	−
5	47	Dyspnea	Depression	−	+	None	None	65.3	55.7	22.8	22.1	+
6	62	Anterior chest pain	None	−	+	None	None	67.9	77.8	10.5	17	+
7	22	None	Postoperative PE	−	−	None	RAL,LAL	56.5	91.2	6.3	27.3	−
8	19	None	Depression	−	−	None	LAL	94.6	82.3	10.2	3	+
9	16	None	None	−	−	None	RVH	57.9	92.1	26.3	35.8	+

Abbreviations: UCG, ultrasonic echocardiography; ECG, electrocardiogram; HI-CT, Haller Index on computed tomography; %VC, % vital capacity; FEV1.0%, forced expiratory volume in one second; DM, diabetes mellitus; Af, atrial fibrillation; PE, pectus excavatum; PH, Pulmonary hypertension; RVH, right ventricular hypertrophy; LVH, left ventricular hypertrophy, RAL, right atrium load; LAL, left atrium load.

**Table 2:** Perioperative results for nine cases of the CRN procedure.

Variable	Count
Median operative time (min)	223 (range, 154–307)
Median intraoperative bleeding (g)	165 (range, 10–904)
Median number of pectus bars	2 (range, 1–3)
Median chest tube duration (day)	4 (range, 2–5)
Median hospital stay (day)	8 (range, 6–37)
Median postoperative HI–CT	3.29 (range, 2.72–4.96)
Post operative complication (*n* = 3)	
SSI	2
Wound seroma	1

Abbreviations: HI-CT, Haller Index on computed tomography; SSI, surgical site infection.

## Discussion

All nine patients underwent the CRN procedure. In general, during the Nuss procedure, the pectus bar must be carefully passed through the anterior mediastinal space under thoracoscopic guidance [2]. In past reports, life-threatening complications and mortality of PE surgery were described [2, 4]. It is important to use transections of the costal cartilage to facilitate sternal elevation and prevent life-threatening complications. In addition, transection is also necessary in dealing with bone stiffness, which is an important factor that affects the lifting effect in PE repair, especially for adults. As reported in the literature, the benefit of the Nuss procedure is that it is less invasive [2]. In 1998, Nuss *et al*. described a thoracoscopic surgery for elevating the sternum performed by using a correction bar that maintained the extensibility of the chest. Chest wall instability after the Ravitch procedure may cause paradoxical breathing with respiratory failure and pseudo-articulation. The benefit of the Nuss procedure in the CRN procedure is that not only is the sternum physically lifted from the interior of the chest cavity using a pectus bar, but also the unstable anterior chest wall is reinforced simultaneously. In addition, the CRN procedure is effective for recurrent cases where intrathoracic adhesions make surgery more difficult. The CRN procedure can be applied to the thoracic cavity and mediastinum to avoid serious complications such as hemothorax, major vessel damage, cardiac, or lung injuries.

There is a lack of consensus regarding whether our surgical correction of PE can improve cardiopulmonary dysfunction [5–9]. To the best of our knowledge, our case with severe stenosis of the pulmonary artery is the first report of PE correction using CRN that has resulted in an increase in pulmonary artery diameter, improvement of pulmonary hypertension, and symptomatic alleviation. Chao *et al.* reported that PE correction led to postoperative structural and functional improvement of the right heart due to the physical relief of cardiac compression, as measured by transesophageal echography.^5^ In our series, improvements in symptoms of respiratory distress and palpitations were also observed in other cases, which confirmed the usefulness of CRN for severe PE with symptoms.

We performed the CRN procedure for severe PE in nine patients without life-threatening complications. In our series, two patients had surgical site infection that did not require removal of the pectus bar and one patient had wound seroma. Although statistical considerations are not possible because of the small number of cases, perioperative complications may include high intraoperative blood loss. Elderly patients with brittle bone or a case of recurrence make the surgical procedure more complicated, which may increase the amount of intraoperative bleeding and increase the rate of SSI. Although the CRN procedure is slightly more invasive in terms of operation time or intraoperative bleeding than the Nuss procedure, there are no significant differences in perioperative management in the duration of chest tube usage, pain management, or length of hospital stay.

The CRN procedure also has problems that need to be addressed. First, it is unclear how to determine which patients should undergo the CRN procedure. We performed this procedure for severe PE with a CT Haller index >10, in cases with severe stenosis of the central pulmonary blood vessel and bronchus causing cardiopulmonary dysfunction, and in a recurrent case. However, in the future, we should examine additional cases and determine the adaptation of this procedure based on objective findings. In addition, the correct timing of bar removal and the long-term prognosis are unknown. Generally, the pectus bar can be safely and easily removed at 2 years or longer after the Nuss procedure and there is no recurrence during the follow-up period [9–12]. However, the CRN procedure that requires re-suturing of the sternum and the costal cartilage may need longer fixation duration than the timing of bar removal in the Nuss procedure. Therefore, although there is no recurrence in this study, careful postoperative observation is warranted.

In summary, these cases suggest that the surgical repair of severe and asymmetrical PE with cardiopulmonary dysfunctions can significantly improve not only a patient’s appearance but also a patient’s symptoms. The CRN procedure we have developed is an effective and safe technique for severe PE.
